# Ursolic Acid Ameliorates Myocardial Ischaemia/Reperfusion Injury by Improving Mitochondrial Function via Immunoproteasome-PP2A-AMPK Signalling

**DOI:** 10.3390/nu15041049

**Published:** 2023-02-20

**Authors:** Luo-Luo Xu, Hui-Xiang Su, Pang-Bo Li, Hui-Hua Li

**Affiliations:** Beijing Key Laboratory of Cardiopulmonary Cerebral Resuscitation, Department of Emergency Medicine, Beijing Chao-Yang Hospital, Capital Medical University, No. 8 Worker’s Stadium South Road, Beijing 100020, China

**Keywords:** myocardial ischaemia/reperfusion, ursolic acid, immunoproteasome, mitochondrial function, PP2A, AMPK

## Abstract

Cardiac ischaemia/reperfusion (I/R) injury causes cardiomyocyte apoptosis and mitochondrial dysfunction. Ursolic acid (UA), as a pentacyclic triterpenoid carboxylic acid, exerts several bioactivities in animal models of different diseases, but the preventive role of UA in I/R-induced myocardial dysfunction remains largely unknown. Male wild-type mice were pre-administered with UA at a dosage of 80 mg/kg i.p. and then subjected to cardiac I/R injury for 24 h. Cardiac function and pathological changes were examined by echocardiography and histological staining. The protein and mRNA levels of the genes were determined using qPCR and immunoblotting analysis. Our results revealed that UA administration in mice significantly attenuated the I/R-induced decline in cardiac function, infarct size, myocyte apoptosis, and oxidative stress. Mechanistically, UA increased three immunoproteasome catalytic subunit expressions and activities, which promoted ubiquitinated PP2A degradation and activated AMPK-PGC1α signalling, leading to improved mitochondrial biosynthesis and dynamic balance. In vitro experiments confirmed that UA treatment prevented hypoxia/reperfusion (H/R)-induced cardiomyocyte apoptosis and mitochondrial dysfunction through activation of AMPK signalling. In summary, our findings identify UA as a new activator of the immunoproteasome that exerts a protective role in I/R-induced myocardial dysfunction and suggest that UA supplementation could be beneficial for the prevention of cardiac ischaemic disease.

## 1. Introduction

Myocardial infarction (MI) is a well-known medical disease that causes high morbidity and mortality rates in the world. Timely reperfusion has been demonstrated to efficiently limit infarct size and attenuate cardiac dysfunction, but it also aggravates cardiac tissue injury, which is known as cardiac ischaemia and reperfusion (I/R) injury [1]. Interestingly, multiple pathophysiological features are involved in the pathological process of cardiac I/R impairment, including cell death, inflammation, Ca^2+^ overload, excessive production of oxygen free radicals (ROS), endothelial dysfunction, aggregation of platelets, and mitochondrial energy dysfunction [1]. However, at present, no available treatments effectively protect the heart against this injury. Thus, it is essential to discover and develop novel strategies to prevent or cure myocardial I/R injury to ameliorate clinical outcomes in MI patients.

The proteasome complex is a proteolytic enzyme that regulates the degradation of misfolded, damaged, or aggregated proteins in mammalian cells, which is required to maintain the homeostasis of proteomes and most cellular processes [2]. The core 20S proteasome is composed of 28 α or β subunits. Among them, there are three constitutive catalytic β subunits, including β1, β2, and β5 (also known as PSMB6, PSMB7, and PSMB5, respectively), which are responsible for three proteolytic activities (caspase-like, trypsin-like, and chymotrypsin-like, respectively). Upon inflammatory stimulation, these constitutive β subunits are substituted by three β immunoprotease subunits, β1i, β2i, and β5i (also known as PSMB9/LMP2; PSMB10/LMP10; PSMB8/LMP7), to generate the 20S immunoproteasome [2,3]. Early studies indicated that the immunoproteasome primarily regulates antigen presentation and the inflammatory response [2]. Recently, more studies have demonstrated that the expression and activity of the β2i and β5i subunits are highly upregulated during heart angiotensin II infusion or pressure overload [4,5,6,7]; furthermore, these subunits are critically involved in several types of cardiovascular diseases, including heart failure, cardiac hypertrophic remodelling, abdominal aortic aneurysm, and atrial fibrillation [4,5,6,7]. Interestingly, the immunoproteasome plays an important role in the regulation of cardiac ischaemic injury. Activation of the β1i subunit and proteasome activator PA28α restores I/R-mediated cardiac dysfunction by decreasing PTEN protein levels and inhibiting the Akt or HIF-1α signalling pathway [8,9,10]. In contrast, inhibition of endogenous β5 and proteasome chymotrypsin-like activity in mice by overexpressing T60A-β5 significantly accelerates cardiac I/R injury [11]. Thus, an increase in immunoproteasome activity may protect against cardiac I/R injury.

**Figure 1:**
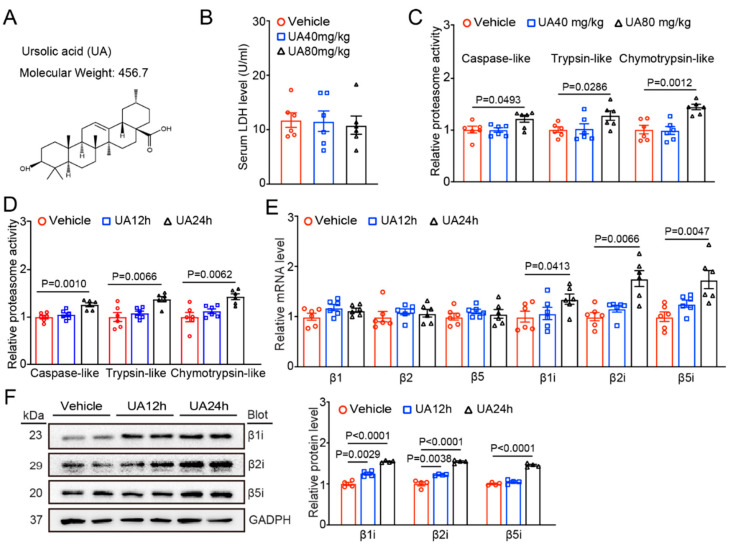
Administration of UA increases the immunoproteasome catalytic subunit activity and expression. (**A**) Chemical structure and molecular weight of UA. (**B**) Male mice (*n* = 6 per group) were administered with UA (40 or 80 mg/kg) for 24 h, serum LDH activity was measured in mice (*n* = 6 per group). (**C**) Analysis of three immunoproteasome activity types in the heart tissues of mice at 24 h after UA administration (40 or 80 mg/kg) (*n* = 6 per group). (**D**) Measurement of three immunoproteasome activity types in cardiac tissue of mice at 12 or 24 h after UA (80 mg/kg) treatment (*n* = 6 per group). (**E**) The mRNA levels of six catalytic subunits of the proteasome in the heart tissues were detected by qPCR analysis (*n* = 6 per group). (**F**) The protein levels of the β1i, β2i, and β5i subunits in the heart tissues were detected by immunoblot analysis (left) and quantification of the relative protein intensities (right, *n* = 4 per group). GAPDH was used as an internal control.

Accumulating evidence from clinical trials and population studies reveals that a healthy diet that is higher in plant foods (whole grains, nuts, seeds, fresh vegetables, and fruits) and lower in animal foods can significantly reduce the risks of noncommunicable diseases, including cancer and cardiovascular disease [12]. Indeed, natural therapeutic agents, which are isolated from plants and vegetables containing active ingredients, can effectively prevent cardiac I/R injury in different models of animals [13]. However, translation into clinical practice is less successful. Ursolic acid (UA; 3 β-hydroxy-urs-12-en-28-oic acid) is a pentacyclic triterpenoid (PT) carboxylic acid which has been identified in numerous traditional medicinal herbs and foods, including ginseng, apple peel, cranberry, plum, calendula, rosemary, and pear [14]. The chemical formula of UA is C_30_H_48_O_3_, with a molecular weight of 456.7 g/mol and low solubility in water (Figure 1A) [14]. UA and its derivatives are safe and have low toxicity against normal cells; furthermore, these compounds have numerous critical pharmacological activities which make them potential therapeutic drugs for cancers [14]. Numerous studies have indicated that UA and its derivatives display diverse biological properties and exert anti-inflammatory, antioxidant, and neuroprotective activities and anti-diabetic effects through different mechanisms [15]. In terms of the antitumour effects, UA can modulate the growth factor receptors (EGFR, HER-2, and PDGF), transcription factors (AP-1, STAT3, p53, and NF-kB), production of pro-inflammatory cytokines, and other molecular mediators which are crucial for the regulation of the cell death, proliferation, metastasis, and angiogenesis of tumour cells [16]. Indeed, UA has a beneficial role in various diseases, such as cancer, diabetes, and some cardiovascular diseases, in experimental animal models. Moreover, several clinical trials have been reported to test the effects of various formulations of UA on healthy subjects or patients with different cancers [15]. The findings from these studies suggest that UA and its distinct derivatives may become potential therapeutic drugs for treating these diseases. Moreover, UA has recently been reported to alleviate apoptosis in hypoxia/reoxygenation (H/R)-induced cardiomyocytes in vitro [17] and ameliorates I/R-mediated myocardial injury in isolated rat heart [18]. Interestingly, a recent study suggests that UA can induce the proteasome activity in Caenorhabditis elegans [19]. However, whether UA could increase proteasome activity in the heart to protect against cardiac I/R-related dysfunction and its underlying mechanism are unclear.

Here, our results indicated that UA exerted a cardioprotective role in I/R-induced cardiac impairment and heart failure. Mechanistically, UA highly upregulated immunoproteasome subunit expression and activity that promoted PP2A degradation and activated AMPK-PGC1α signalling leading to Mfn1/2 and Drp1 balance, thereby improving I/R-induced perturbation of mitochondrial biosynthesis and dynamics. Therefore, our data provide new evidence that UA may be considered a promising candidate for preventing cardiac I/R injury in patients.

## 2. Materials and Methods

### 2.1. Mice

C57BL/6J mice were purchased from Beijing Vital River Laboratory Animal Technology Co., Ltd (Beijing, China). All animals were kept in a pathogen-free room with a temperature of 25 ± 1 °C. Standard mouse chow and water ad libitum were provided to all mice throughout the study in the animal facility of the Beijing Chaoyang Hospital Medical Research Center.

### 2.2. Ischaemia/Reperfusion (I/R) Model and UA Treatment

**Figure 2:**
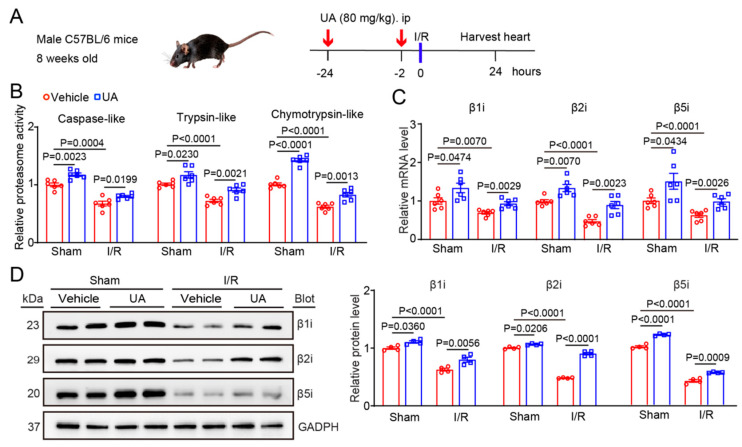
UA enhances the immunoproteasome catalytic subunit expression and activities in the I/R heart tissues. (**A**) Schematic diagram of the experimental design. (**B**) Male mice (*n* = 6 per group) were pretreated with UA (80 mg/kg) before sham or I/R surgery. After 24 h treatment, 3 immunoproteasome activity types in the cardiac tissues of mice were measured (*n* = 6 per group). (**C**) The mRNA levels of the β1i, β2i, and β5i subunits (β1i, β2i, and β5i) in the heart tissue were detected by qPCR analyses (*n* = 6 per group). (**D**) The protein levels of the β1i, β2i, and β5i subunits in the heart tissue were examined by immunoblot analysis (left) and quantification of the relative protein intensities (right, *n* = 4 per group). GAPDH was used as an internal control.

Cardiac I/R injury was induced in male mice (20–23 g, *n* = 46) at 8–9 weeks old by occlusion of the proximal left anterior descending coronary artery (LAD) for 0.5 h followed by reperfusion for 24 h as described previously [20,21]. Sham mice (*n* = 54) underwent the same operation procedure with no LAD artery ligation. To evaluate the impact of UA on cardioprotection against I/R injury in mice, UA (purity = 99.66%; HY-NO140, MCE, powder) was fully dissolved in 5% dimethyl sulfoxide (DMSO) and then diluted with corn oil to reduce DMSO toxicity. A previous study administered UA at 40 mg/kg body weight/day to rats from 3–9 days after isoproterenol injection [22]. Another study administered UA (80 mg/kg/day) for 7 days prior to intraperitoneal (i.p.) injection of doxorubicin in mice [23]. In the present experiment, we chose intraperitoneal administration of UA at 80 mg/kg/day (200 μL per mouse) to mice 2 and 24 h prior to the I/R operation based on our preliminary data shown in Figure 1 and Figure 2. Corn oil without UA was administered to mice as vehicle control. To test the impact of UA on cardiac I/R damage, animals (*n* = 6 per group) were randomly divided into 4 groups, sham + vehicle, sham + UA (80 mg/kg), I/R + vehicle, and I/R + UA (80 mg/kg).

### 2.3. Echocardiographic Assessment

After 24 h of I/R or sham injury, all mice (*n* = 6 per group) were anaesthetized using 1.5–2.0% isoflurane. Echocardiography was used to detect cardiac structure and left ventricular (LV) function with a Vevo 2100 Imaging System (Visual Sonics Inc., Toronto, ON, Canada) The parameters, including LV internal dimension at end-diastole (LVIDd) and end-systole (LVIDs), LV posterior wall at end-diastole (LVPWd) and end-systole (LVPWs), LV anterior wall at end-diastole (LVAWd) and end-systole (LVAWs), ejection fraction (EF%), and fractional shortening (FS%) for all mice were analysed as previously described [21,24]. All data used for the calculation of these parameters are provided in Supplementary Table S1.

### 2.4. Evaluation of Cardiac Infarct Size

Measurement of cardiac infarct size was performed at 24 h of reperfusion. Animals (*n* = 6 per group) were anaesthetized with 100 mg/kg pentobarbital sodium. The heart was flushed with 0.9% saline, the LAD artery was religated, and 200 μL of 1% Evans blue solution was infused into the LV. Then, frozen heart was cut into 4 equal sections at 2 mm thickness and then dyed in 1% 2,3,5-TTC solution (Sigma–Aldrich, Saint Louis, MO, USA) for 20 min at 37 °C. After 24 h of fixation in 4% paraformaldehyde, each section was photographed by a Leica microscope (Wetzlar, Germany), and Image-Pro Plus software (National Institutes of Health) was used to analyse all images. The infarct area is shown in red, the LV area at risk (AAR) is shown in white + red, and the nonischaemic region is shown in blue. Average percentage of total ischaemic areas = infarct area/LV area; percentage of AAR = (infarct + at-risk) area/LV area [21].

### 2.5. Histological Examinations

Heart tissues from sham or I/R mice (*n* = 6 per group) were fixed in 4% tissue-fixing fluid (Solarbio) and embedded in paraffin. Four sections of heart tissue were cut serially and adhered to slides. A TUNEL Apoptosis Detection Kit was used to evaluate myocyte apoptosis in the heart sections based on the manufacturer’s description (Roche). Cardiac myocytes were confirmed with fluorescence staining using an antibody against α-actinin (green). DAPI was used to identify nuclei (blue). For detection of reactive oxygen species (ROS) levels, heart tissue was immersed in optimal cutting temperature compound and sliced into 5 µm thick cryosections, which were stained with 1 µmol/L dihydroethidium (DHE) in PBS for 30 min at 37 °C. Each section was visualized using a Leica fluorescence microscope (German). Five visual fields were chosen randomly in each sample for the analysis of TUNEL-positive myocytes or DHE intensity. TUNEL-positive cells as well as ROS levels were analysed by ImageJ software.

### 2.6. Analysis of Proteasome Activity

Three types of proteasome caspase-like, trypsin-like, and chymotrypsin-like activities were detected in the I/R or sham heart tissues (*n* = 6 per group) using 3 fluorogenic peptide substrates (Z-mLPnLD, Z-LRR, and Suc-LLVY) in Proteasome-Glo assay kits (Promega, Madison, WI, USA) as previously described [4,25]. The proteins from ischaemic areas of the heart were purified with PBS. Equal volumes of protein supernatant and prepared proteasome substrate reagent were incubated in a 96-well plate for 10 min (37 °C). The fluorescence intensity for each sample was detected on a multimode microplate reader (Tecan, Spark model).

### 2.7. Cell Culture, Hypoxia/Reoxygenation Model, and Treatment

Neonatal rat cardiac myocytes (NRCMs) were purified from 1-day-old Sprague–Dawley (SD) rats (total 18 rats, *n* = 3 per group), which were sterilized with 75% alcohol. The hearts were quickly removed, cut into small pieces (1 mm^3^), and digested with trypsin and collagenase II based on a previous report [4]. Isolated cardiomyocytes were incubated in DMEM/F12 supplemented with 15% FBS for 24 h and then incubated in serum-free DMEM/F12 containing the appropriate chemicals for subsequent in vitro studies.

For the hypoxia/reoxygenation (H/R) assay in vitro, NRCMs were incubated in hypoxia buffer containing NaCl, NaHCO_3_, NaH_2_PO_4_·2H_2_O, anhydrous CaCl_2_, MgCl_2_·6H_2_O, sodium lactate, KCl, 2-D-ribose, and 2-deoxyglucose under hypoxic conditions (1% O_2_) for 6 h, and then the cells were incubated in normal DMEM (without FBS) and 1% penicillin/streptomycin and reoxygenated under normal oxygen conditions (95% O_2_) for 24 h. UA (0.5 µM) and Compound C (CC, an AMPK inhibitor, 10 µM) or both were added to the culture medium for 24 h prior to H/R injury as described previously [26,27,28].

### 2.8. Mitochondrial and TUNEL Staining In Vitro

Mitochondria in cardiomyocytes were stained using 0.02 μM MitoTracker Red CMXRos (Beyotime) fluorescent probe for 20 min based on the instructions provided by the manufacturer. Mitochondrial images were photographed using a confocal microscope (Zeiss LSM510 META) as previously described [29]. Five visual fields were randomly selected from each group to quantify the number of mitochondrial fission in each image to obtain the mean value of each sample (*n* = 3/group).

For apoptosis measurement of NRCMs (*n* = 3 per group), cells cultured in 96-well plates were exposed to H/R conditions treated with and without UA (0.5 µM). After fixation with 4% paraformaldehyde and 0.2% Triton X-100 in PBS, cells were stained with a TUNEL Apoptosis (red) Detection Kit and DAPI (blue) based on the protocols provided by the manufacturer (Roche). Five images in each sample were randomly selected to quantify the number of apoptotic cells.

### 2.9. Mitochondrial Membrane Potential and mPTP Opening Detection In Vitro

For the detection of mitochondrial membrane potential (∆Ψm, MMP), NRCMs were washed with PBS and then stained with JC-1 working solution (Beyotime) for 20 min at 37 °C. Five images of each sample were photographed using fluorescence microscopy (Leica, DM2500) and analysed by ImageJ software (1.48v). The relative ratio of JC-1 aggregate (red)-to-monomer (green) fluorescence intensity was used to evaluate the proportion of depolarized mitochondria [30].

For the examination of mitochondrial permeability transition pore (mPTP), NRCMs were stained with a Mitochondrial Permeability Transition Pore Assay Kit based on the description of manufacturer (Beyotime). The relative calcein green fluorescence intensity in mitochondria was used to judge the degree of mPTP opening. All values of fluorescence intensity were normalized to controls. Five visual fields in each sample were randomly chosen to quantify the JC-1 or mPTP fluorescence intensity.

### 2.10. Examination of ATP Levels

Cardiac ATP levels in each sample (*n* = 6 per group) were examined with an ATP-level assay kit based on the manufacturer’s description (Beyotime). Ten milligrams of fresh cardiac tissues (*n* = 6 per group) was resuspended in ATP lysis buffer (100 μL), homogenized, and centrifuged at 12,000× *g* for 5 min. Then, supernatants were added into ATP assay working solution (100 μL) for 5 min [21,31]. A spectrophotometer plate reader (Tecan, Spark model) was used to measure the fluorescence intensity, and all values were normalized to controls.

### 2.11. LDH Activity Measurement

Supernatant from the fresh heart tissues (*n* = 6 per group) and serum from the mice (*n* = 6 per group) were collected as described in the I/R model method. Lactate dehydrogenase (LDH) activity was measured with an LDH activity assay kit (Jiancheng Bioengineering Institute, Nanjing, China). The fluorescence intensity for each sample was recorded at an excitation wavelength of 450 nm.

### 2.12. Quantitative Real-Time PCR Analysis

Sham or I/R hearts (*n* = 6 per group) were harvested. Total RNA was isolated from the border zone of the heart with TRIzol reagent (Takara). Equal amounts (1–2 μg) of total RNA and RT Enzyme mix (Takara) were used to synthesize cDNA. The mRNA expression of the target genes was analysed with a PCR thermocycler (Applied Biosystems) as previously described [21]. The data were normalized to GAPDH expression levels. All PCR primer sequences for each gene used are provided in Table 1.

### 2.13. Immunoblotting Analysis

The I/R or sham hearts (*n* = 4 per group) were flushed with PBS and harvested. The ischaemic border zone of the heart was lysed with RIPA lysis buffer containing protease inhibitors and sonicated as described [21]. The protein concentrations of each sample were measured with a protein assay kit based on the instructions of the manufacturer. Equal amounts (40–50 μg) of proteins were separated by SDS–PAGE on 10% gels, transferred to PVDF membranes, and then incubated with the primary and secondary antibodies. Each blot density was quantified with scanning densitometry using a FluorChem R (ProteinSimple) imaging system and normalized to GAPDH as previously described [21]. All primary antibodies used in this study are shown in Supplementary Table S2.

### 2.14. Immunoprecipitation and Ubiquitylation Assays

Cardiac tissues were lysed with lysis buffer containing 20 mM trisHCl (pH 7.5), 150 mM NaCl, 1% Triton X-100, sodium pyrophosphate, β-glycerophosphate, EDTA, Na_3_VO_4_, leupeptin, and plus PMSF (Beyotime) on ice for 0.5 h. The cell lysates were centrifuged at 12,000 rpm for 15 min (4 °C) to obtain the protein supernatants. Then, 2 mg/mL of the protein was incubated with anti-PP2A rabbit antibody (0.33 μg) and 30 μL of protein A–Sepharose (Amersham Biosciences), and gently shaken at 4 °C for 24 h. The pellets were washed five times with wash buffer (Beyotime). Bound proteins were eluted with 2× sample buffer with boiling. The ubiquitinated PP2A protein was evaluated with immunoblotting analysis with an anti-ubiquitin (Ub) antibody [4].

### 2.15. Statistical Analysis

All results are expressed as the mean ± SEM. Statistical analyses were carried out with GraphPad Prism 9 or IBM SPSS Statistics software 21.0. The Shapiro–Wilk normality test was used to examine the normal distribution of the data. Differences among groups were tested by one-way ANOVA analysis. A value of *p* < 0.05 was considered statistically significant.

## 3. Results

### 3.1. UA Upregulates Cardiac Immunoproteasome Subunit Expression and Activity

UA has been reported to be an inducer of proteasome activity in the brain [19]. To determine whether UA activates proteasome activity in the heart, we treated WT mice with two doses (40 or 80 mg/kg) of UA for 24 h. Compared with the vehicle control, UA treatment at both 40 and 80 mg/kg had no cardiotoxic effect, as reflected by the measurement of LDH activity (Figure 1B). However, UA dose-dependently increased the three catalytic caspase-like, trypsin-like, and chymotrypsin-like activities of the proteasome in the heart tissues, similar to what has been previously described [4,6,25,32] (Figure 1C). Therefore, we chose UA at 80 mg/kg to treat mice for 12 or 24 h and found that compared with the vehicle control, UA treatment for 24 h markedly upregulated the activity of all three proteasome subunits in the heart (Figure 1D). Next, we determined which catalytic subunits of the proteasome were responsible for the increased proteasome activity. qPCR analysis showed that among the six catalytic subunits, the mRNA levels of the β1i, β2i, and β5i subunits, but not those of the β1, β2, and β5 subunits, were highly enhanced after 24 h of UA treatment (Figure 1E). The increased protein levels of β1i, β2i, and β5i were confirmed in cardiac tissue by immunoblotting analysis (Figure 1F). Taken together, these data reveal that UA at 80 mg/kg effectively enhances cardiac immunoproteasome activity via upregulation of the inducible catalytic subunits.

### 3.2. UA Attenuates the I/R-Mediated Reductions in Cardiac Immunosubunit Expression and Activity

To test whether UA prevents the development of I/R-triggered cardiac injury by upregulating proteasome activity, we treated WT mice with UA (80 mg/kg) for 24 h before I/R surgery (Figure 2A). Consistent with a previous report [11], I/R for 24 h significantly reduced three caspase-like, trypsin-like, and chymotrypsin-like activities in the heart tissues of the I/R model mice compared with those in the hearts of the sham mice, whereas this decrease was markedly blunted in the hearts of UA-treated I/R model mice (Figure 2B). Accordingly, I/R-mediated decreases in the mRNA and protein levels of the β1i, β2i, and β5i subunits were also reversed in the hearts of UA-treated mice (Figure 2C,D). Moreover, UA treatment also increased the β1i, β2i, and β5i expression and activities under sham conditions (Figure 2A–D).

### 3.3. UA Ameliorates I/R-Triggered Cardiac Impairment and Dysfunction

**Figure 3:**
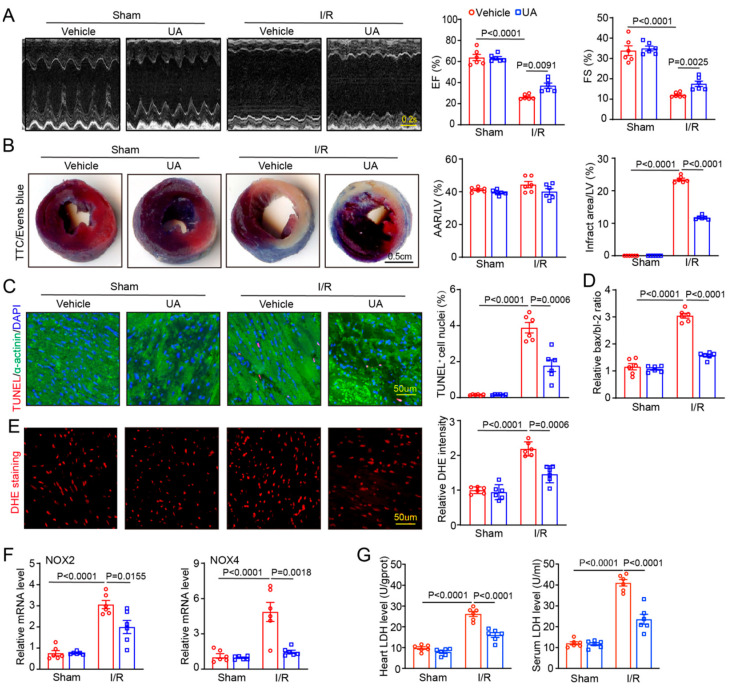
Administration of UA alleviates I/R-triggered cardiac injury and dysfunction. (**A**) Male mice (*n* = 6 per group) were pretreated with UA (80 mg/kg) and then exposed to sham or I/R surgery for 24 h. Representative M-mode echocardiographic images of the LV (left). Scale bar: 0.2 s. Quantification of EF% or FS% in each group (right, *n* = 6 per group). (**B**) Representative images of TTC/Evans blue staining of heart sections (left). The ratios of area at risk (AAR)/LV area and infarct size/AAR (right, *n* = 6 per group). (**C**) Representative images of TUNEL (red), α-actinin (green), and DAPI (blue) staining of the heart sections and percentage of TUNEL-positive nuclei (*n* = 6 per group). (**D**) The mRNA levels of Bax and Bcl-2 were detected by qPCR analysis. The results are presented as the Bax/Bcl-2 ratio (*n* = 6 per group). (**E**) DHE staining of heart sections (left) and quantification of the DHE fluorescence intensity for ROS level (right, *n* = 6 per group). Scale bar: 50 μm. (**F**) The mRNA levels of NOX2 and NOX4 were detected by qPCR analysis (*n* = 6 per group). (**G**) Measurement of LDH activity in serum and heart tissues (*n* = 6 per group).

To determine whether UA-enhanced proteasome activity prevents I/R-mediated cardiac dysfunction in vivo, mice were pre-treated with UA (80 mg/kg) before sham or I/R surgery. After 24 h treatment, echocardiography indicated that I/R resulted in a significant decline in cardiac contractile function as indicated by reduced EF% and FS%, in vehicle-treated mice compared with sham mice, but this decrease was remarkably attenuated in UA-treated mice (Figure 3A, Supplementary Table S1). Accordingly, I/R-mediated enlargement of left ventricular (LV) chamber dimensions as indicated by increased LVIDd and LVIDs, was also greatly reduced in UA-treated mice (Supplementary Table S1). Then, we assessed the action of UA on I/R-mediated cardiac infarct size and cell apoptosis, which are the main inducers of cardiac injury and dysfunction. TTC/Evans blue and TUNEL staining revealed that I/R injury for 24 h highly augmented the infarct size as indicated by the increased infarct area/LV ratio, percentage of TUNEL^+^ myocytes, and Bax/Bcl-2 ratio in vehicle-treated mice compared with sham mice, but these actions were all dramatically suppressed in UA-treated mice after I/R injury (Figure 3B,C). Moreover, the ROS burst from the mitochondrial complex is a critical cause of cardiac I/R injury. We then tested the impact of UA treatment on oxidative stress and found that it markedly suppressed the I/R-mediated increase in the ROS level as indicated by increased DHE fluorescence intensity, the mRNA expressions of NADPH isoforms NOX2 and NOX4, as well as the LDH activity in serum and heart tissues compared with vehicle treatment after I/R injury (Figure 3D–G). Thus, these data suggest that UA has a cardioprotective role against I/R impairment.

### 3.4. UA Promotes Mitochondrial Biogenesis and Dynamic Balance through Activation of AMPK-PGC1α Signalling and Increased PP2A Degradation

**Figure 4:**
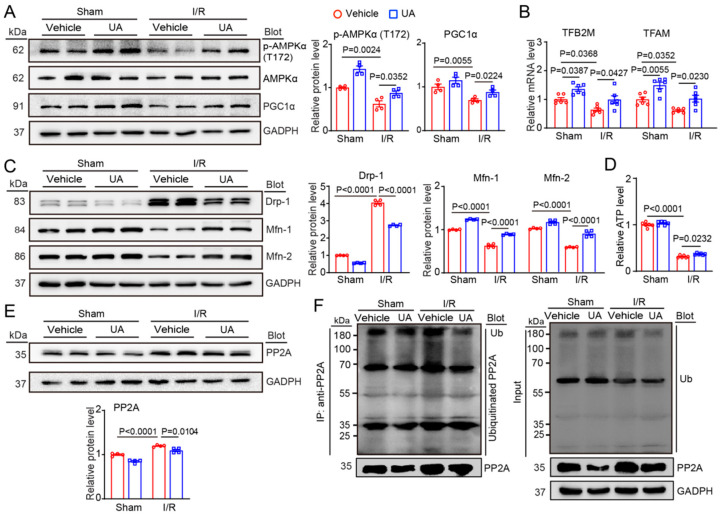
UA promotes mitochondrial biogenesis and dynamic balance via AMPK–PGC1α-activated AMPK signalling in the I/R heart. (**A**) Male mice (*n* = 6 per group) were pretreated with UA (80 mg/kg) and then exposed to I/R for 24 h. Immunoblotting analysis of phosphorylated (*p*)-AMPKα (T172), total AMPKα, and PGC1α proteins in the heart tissue (left) and quantification of relative *p*-AMPKα/AMPKα and PGC1α protein levels (right, *n* = 4 per group). (**B**) qPCR analysis of TFAM and TFB2M (*n* = 6 per group). (**C**) Immunoblot analysis of Drp1 and Mfn1/2 proteins in heart tissue (left) and analysis of the relative protein intensities (right, *n* = 4 per group). GAPDH is an internal control. (**D**) Measurement of relative ATP levels (*n* = 6 per group). (**E**) Immunoblot analysis of PP2A protein in heart tissue (top) and analysis of the relative protein intensities (bottom, *n* = 4 per group). (**F**) Lysates from heart tissue pretreated with vehicle or UA after sham or I/R operation before harvest were used for immunoprecipitation with anti-PP2A antibody. The ubiquitinated PP2A was evaluated by immunoblotting analysis with anti-ubiquitin (Ub, upper left) and anti-PP2A antibody (lower left). Input showed the protein levels of Ub and PP2A in heart lysates (right).

Mitochondrial dysfunction has been considered a critical mechanism of cardiac I/R damage, and the AMPK axis exerts a key role in regulating mitochondrial biogenesis and dynamic balance during I/R injury [33]. Therefore, we tested the impact of UA on the activation of AMPK, regulators of mitochondrial biogenesis (PGC1α, TFAM, and TFB2M), and mitochondrial dynamics (Drp1 and Mfn1/2). Immunoblotting and qPCR analysis indicated that I/R surgery greatly reduced the protein levels of phosphorylated (*p*)-AMPKα (T172) and total PGC1α and the mRNA levels of TFAM and TFB2M in mice compared to those in the sham control, whereas this decrease was significantly attenuated in UA-treated mice (Figure 4A,B). Similarly, UA treatment also increased the protein levels of (*p*)-AMPKα (T172) and PGC1α and the mRNA levels of TFB2M and TFAM after sham surgery (Figure 4A,B). Moreover, I/R surgery-induced upregulation of the pro-fission protein Drp1 and downregulation of the profusion proteins Mfn1/2, which were greatly reversed in the hearts of UA-treated mice after I/R (Figure 4C). In addition, the ATP level was significantly higher in the hearts of UA-treated mice than in the hearts of vehicle-treated mice after I/R injury (Figure 4D). Therefore, these data suggest that UA treatment can improve cardiac mitochondrial biogenesis and mitochondrial dynamic imbalance after I/R surgery.

Multiple studies have revealed that activation of AMPK signalling is negatively regulated by PP2A, a Ser/Thr phosphatase [34]. To understand the molecular mechanisms whereby UA activates the AMPK-PGC1α axis in the I/R heart, we tested the effect of UA on cardiac PP2A protein levels. We found that I/R surgery significantly upregulated PP2A protein levels in vehicle-treated mice, an effect that was markedly reversed in UA-treated mice (Figure 4E). This effect likely occurred because PP2A stability is known to be modulated by the ubiquitin-proteasome system [35,36,37]. Consistent with these findings, the immunoprecipitation assay confirmed that I/R caused a significant upregulation of ubiquitinated PP2A proteins due to reduced proteasome activity, but this increase was blocked in UA-treated hearts (Figure 4F).

### 3.5. UA Improves H/R-Induced Cardiomyocyte Apoptosis, Mitochondrial Fragmentation and Dysfunction, Whereas Inhibiting AMPK Abolishes These Effects In Vitro

**Figure 5:**
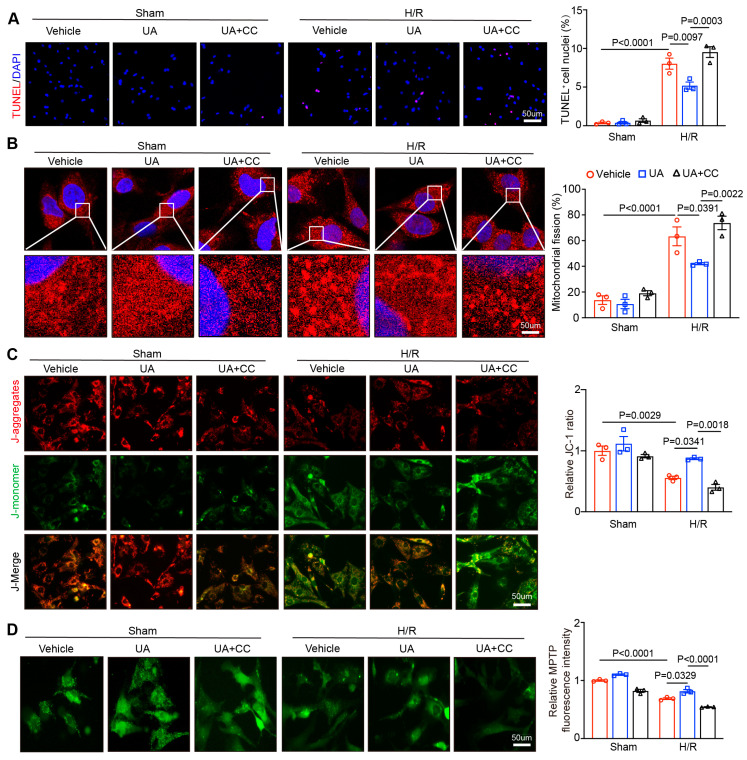
Inhibiting AMPK activation abolishes the UA-mediated protective effect against H/R-triggered cardiomyocyte apoptosis, mitochondrial fragmentation, and mitochondrial dysfunction in vitro. (**A**) NRCMs were cotreated with CC (an AMPK inhibitor; 10 µM) and UA (0.5 µM) and then subjected to H/R for 24 h. TUNEL (red) and DAPI (blue) staining of NRCMs (left). Five images from each sample were randomly selected to calculate the apoptotic cells (right, *n* = 3 independent experiments). (**B**) Immunofluorescence staining of mitochondrial morphology in NRCMs was performed (left). The images were captured using confocal microscopy. Five images from each sample were randomly selected to calculate the percentage of mitochondrial fission in each image to obtain the mean value of each sample (right, *n* = 3 independent experiments). (**C**) JC-1 fluorescence staining of NRCMs to determine the ΔΨm. Red: high potential (J-aggregates); green: low potential (J-monomers). The images were visualized using fluorescence microscopy. Five visual fields were selected randomly from each sample to analyse the JC-1 intensity (right, *n* = 3 independent experiments). (**D**) Fluorescence staining of NRCMs to detect mPTP opening (left). The images were visualized using fluorescence microscopy. Five visual fields were selected randomly from each sample to analyse the mPTP intensity (right, *n* = 3 independent experiments). Scale bar: 50 μm.

To validate the cardioprotective role of UA on cardiac I/R injury in vivo, we assessed the impact of UA on H/R-mediated cardiomyocyte apoptosis and mitochondrial function in vitro. Neonatal rat cardiomyocytes (NRCMs) were treated with UA at 0.1–5 µM for 24 h based on previous reports [26,27]. Measurement of LDH activity indicated that UA at 0.5 μM had no toxicity on NRCMs (data not shown). Next, NRCMs were cotreated with UA (0.5 μM) and CC (10 µM) for 2 h and then exposed to sham or H/R condition for an additional 24 h. Immunostaining of NRCMs indicated that H/R exposure for 24 h markedly increased the apoptotic cell numbers and mitochondrial fission and aggravated mitochondrial dysfunction as indicated by increased MMP and mitochondrial permeability transition pore (mPTP) values compared with those of the sham group after vehicle treatment (Figure 5A–D, lane 4 vs. 1). These effects were reversed in UA-treated NRCMs (Figure 5A–D, lane 5 vs. 4), but the reversals were abolished in CC-treated NRCMs (Figure 5A–D, lane 6 vs. 5). However, there was no difference in the parameters for mitochondria after sham treatment (Figure 5A–D, lane 1–3).

## 4. Discussion

Natural vegetation is the main source of active substances that are used as therapeutic drugs for numerous diseases. One group of compounds isolated from plants is PT, which has been reported to have many health benefits, including antiapoptotic, anti-inflammatory, antitumoural, and antibacterial properties with low toxicity [14]. Interestingly, high consumption of various fruits and vegetables is related to a reduced incidence of cancer and diseases in animal models [14]. Moreover, UA and oleanolic acid (OA) are two representative examples of PT compounds and are found in a number of plants. Multiple studies have confirmed that UA exerts antioxidative and antiapoptotic properties, playing protective roles in the development of several cardiovascular conditions, particularly hypertension, atherosclerosis, cardiac toxicity, cardiac remodelling, and myocardial infarction [14,21,22]. More recently, a study suggested that UA treatment significantly ameliorated H/R-mediated apoptosis of cultured H9c2 cardiomyocytes In vitro [17]. In the present study, our results verified these previous findings and further identified that UA, as a bioactive compound, increases immunoproteasome expression and activity, which promotes PP2A degradation and improves AMPK-dependent mitochondrial function, thereby leading to the attenuation of I/R-triggered cardiac dysfunction (Figure 1, Figure 2, Figure 3 and Figure 4). The protective effects of UA against H/R-mediated cardiomyocyte apoptosis and mitochondrial dysfunction were confirmed in cultured primary cardiomyocytes (Figure 5). Therefore, our data indicate that UA can prevent I/R-mediated mitochondrial impairment and cardiac dysfunction possibly by increasing the activity of the immunoproteasome–PP2A–AMPK pathway and highlight that UA supplementation could be beneficial for patients who undergo cardiac I/R injury.

The immunoproteasome is mainly involved in the inflammatory response and its expression and activity are markedly upregulated in both immune and nonimmune cells under various forms of cellular stress. Many inflammatory cytokines (IFN-γ and TNF-α) and other factors (ON, angiotensin II, and pressure overload) can upregulate the expression of the β1i, β2i and β5i subunits in different tissues and cell types via multiple signalling pathways [2,3,4,5,6,38]. In this study, we further identified that UA treatment significantly enhanced the expression levels of β1i, β2i, and β5i subunits and their activities in the hearts of mice (Figure 1) and markedly reversed the I/R-induced reduction in the three immunoproteasome subunits (Figure 2), suggesting that UA is an activator of the immunoproteasome at the transcriptional level. However, the precise mechanism by which UA upregulates immunoproteasome subunit expression remains to be elucidated.

Mitochondrial quality control mechanisms are required for preserving mitochondrial fission/fusion dynamic balance, mitophagy activation, and cell survival, which are key contributors to cardiac I/R impairment. AMPK is a herotrimeric complex composed of α/β/γ subunits and is activated by activated by upstream kinases (LKB1,CaMKK2, and TAK1) and increased ratio of ADP or AMP/ATP but is inhibited by protein phosphatase 2A and 2C (PP2A, PP2C) and oxidation or acetylation of cysteines in the α subunit [39]. AMPK is a key energy sensor that modulates cardiac glucose and fatty acid metabolism and exerts beneficial effects against cardiac I/R impairment through multiple mechanisms, which include amelioration of oxidative stress and inflammation, increase in mitochondrial synthesis and improvement of dynamic balance [40]. Pharmacological activation of AMPK protects against I/R-mediated cardiac myocyte death and contractile dysfunction [39]. AMPK/mTOR and AMPK/ULK1 signalling pathways are also crucial for activation of autophagy/mitophagy, which is a cytoprotective mechanism for cardiac I/R injury [39]. Meanwhile, AMPK critically promotes PGC-1α-dependent mitochondrial biogenesis by activating NRF1/2-MTFA-mediated transcription and replication of mitochondrial DNA in I/R [41,42]. Furthermore, mitochondrial dynamics are tightly controlled by a range of dynamin GTPases, such as Drp1 (the key factor for mitochondrial fission) and Mfn1/2 (key factors for mitochondrial fusion) [43]. The imbalance of Drp1 and Mfn1/2 levels leads to excessive mitochondrial fragmentation, which is an early hallmark of mitochondrial dysfunction and cardiomyocyte death after I/R surgery [43]. Increasing evidence indicates that AMPK is critically involved in regulating mitochondrial dynamics and redox homeostasis by suppressing the Drp1, NOX4, and SIRT1-PGC-1α pathways [44]. Previous reports suggest that UA has anti-inflammatory, antioxidant and antiapoptotic effects by regulating different signalling pathways, including Nrf2, PPARα, Bcl2-BclxL, p53-Bak-caspase-3, AKT-NO, NOX4-ROS, and CXCL2-NF-κB [17,22,23,45,46,47,48]. However, it is unclear whether UA regulates AMPK-PGC1α and subsequent mitochondrial biogenesis and dynamics in the heart after I/R injury. Here, our data revealed that UA significantly inhibited the I/R-mediated decreases in the protein levels of *p*-AMPK and PGC-1α and the mRNA levels of TFAM and TFB2M in heart tissues (Figure 4A,B). Moreover, I/R-induced upregulation of Drp1 and downregulation of Mfn1/2 were effectively reversed in the I/R heart (Figure 4C). Together, these data suggest that UA can reverse I/R-induced impairment of cardiac mitochondrial biogenesis and dynamic balance through AMPK-dependent signalling.

Phosphatases are involved in the cell cycle in tumours and are being actively explored as therapeutic targets. PP2A is a serine/threonine phosphatase that regulates over 50 protein kinases, including MAPKs and AMPK. Studies have demonstrated that PP2A can dephosphorylate Thr172 of the α-subunit of AMPK to inactivate its kinase activity [34,49]. Accumulating evidence suggests that PP2A plays critical roles in regulating various cellular processes and diseases, including cell apoptosis, autophagy, cancer, cardiac I/R injury, and MI, by inhibiting AMPK-dependent pathways [50,51,52,53,54]. Thus, modulating PP2A expression may represent a promising strategy for treating these diseases [49]. Indeed, PP2A activity can be modulated by posttranslational modifications, including phosphorylation, carboxymethylation, and ubiquitination. Several studies have revealed that ubiquitination of PP2A mediated by ubiquitin E3 ligases promotes its degradation by the proteasome. Conversely, knockout of E3 ligases or inhibition of the proteasome increases PP2A protein levels and activity, which thereby regulates cell apoptosis and asthma [35,36,37]. However, it is unknown whether UA-mediated immunoproteasome activity enhances PP2A degradation in cardiomyocytes after I/R injury. Our data revealed that UA treatment could significantly increase I(H)/R-induced degradation of PP2A, leading to the activation of AMPK-PGC1α-dependent signalling pathways in the heart and cultured cardiomyocytes (Figure 4E). Similarly, the immunoprecipitation assay confirmed that I/R significantly increased ubiquitinated PP2A protein levels due to reduced immunoproteasome activity, but this effect was reversed in the hearts of UA-treated mice (Figure 4F). These data suggest that UA can promote PP2A degradation possibly by increasing immunoproteasome activity.

## 5. Conclusions

This study revealed that UA exerts a critical role in protecting against I/R-induced cardiac injury and dysfunction. UA treatment markedly upregulated the immunoproteasome subunit expression and activity, which increased PP2A degradation that led to activation of AMPK-PGC1α signalling and the Drp1/Mfn1/2 balance, thereby improving mitochondrial biosynthesis and dynamic balance in the I/R heart. Our data also highlight that UA supplementation could be a promising strategy for the prevention of cardiac ischaemic disease. Future studies need to confirm the protective role of UA in other animal models of cardiac I/R injury, to elucidate the potential mechanism by which UA upregulates immunoproteasome activity to promote PP2A degradation in the I/R heart, and to define whether supplementation with UA could be a new option for preventing I/R-related diseases.

## Figures and Tables

**Table 1:** List of primers used in quantitative real-time PCR analysis.

Gene	Forward (5′-3′)	Reverse (5′-3′)
β1 (PSMB6)	TCACTGCCAATGTGTCCTCG	CGTGGCAATGGTGAACTTGG
β1i (PSMB9)	AAGTCCACACCGGGACAAC	TCCCAGGATGACTCGATGGT
β2 (PSMB7)	CTCGGCCCGGAAACACTTT	CAAGACAGCATTCCTGCGAC
β2i (PAMB10)	GACAAAAGCTGCGAGAAGATCC	CGCGTAGTCATCTCAAGTGTCC
β5 (PSMB5)	CCTGGCCTTCAAGTTTCTCCA	CGACCGAGATGCGTTCCTTA
β5i (PSMB8)	GCCAAGGAGTGCAGGTTGTAT	CCAAGGTCGTAGGCCTCTTC
Bax	TGGAGCTGCAGAGGATGATT	CTTGGATCCAGACAAGCAGC
Bcl-2	CAGCCTGAGACAACCCAAT	TATAGTTCCACAAAGGCATCCCAG
NOX-2	GGGAACTGGGCTGTGAATGA	CAATTGTGTGGATGGCGGTG
NOX-4	CCAAATGTTGGGCGATTGTGT	GCCATCGTTTCTGACAGAGC
TRAM	GGGAATGTGGAGCGTGCTAA	ACTTCGGAATACAGACAAGACTGA
TFB2M	GGTCCTGGAATCCTGACTGG	TCCTCTGTAAGGGCTCCAAA
GADPH	GAAGGTCGGTGTGAACGGAT	ACTGTGCCGTTGAATTTGCC

## Data Availability

Not applicable.

## Supplementary Materials

The following supporting information can be downloaded at: https://www.mdpi.com/article/10.3390/nu15041049/s1, Table S1: Echocardiographic measurement of wild type (WT) mice with Ursolic acid injection subjected to I/R model for 24 h. Table S2: List of primary antibodies used in immunoblotting analysis.
