# Correction: Xu et al. Ursolic Acid Ameliorates Myocardial Ischaemia/Reperfusion Injury by Improving Mitochondrial Function via Immunoproteasome-PP2A-AMPK Signalling. *Nutrients* 2023, *15*, 1049

**DOI:** 10.3390/nu17203247

**Published:** 2025-10-16

**Authors:** Luo-Luo Xu, Hui-Xiang Su, Pang-Bo Li, Hui-Hua Li

**Affiliations:** Beijing Key Laboratory of Cardiopulmonary Cerebral Resuscitation, Department of Emergency Medicine, Beijing Chao-Yang Hospital, Capital Medical University, No. 8 Worker’s Stadium South Road, Beijing 100020, China

In the original publication [1], there was a mistake in Figure 5. During paper submission, in Figure 5D, a mismatch error in the H/R + Vehicle group, took place. The corrected Figure 5D appears below. The authors state that the scientific conclusions are unaffected. This correction was approved by the Academic Editor. The original publication has also been updated.



## References

[1] Xu L.-L., Su H.-X., Li P.-B., Li H.-H. (2023). Ursolic Acid Ameliorates Myocardial Ischaemia/Reperfusion Injury by Improving Mitochondrial Function via Immunoproteasome-PP2A-AMPK Signalling. Nutrients.

