# Supplementary material for: Ursolic Acid Ameliorates Myocardial Ischaemia/Reperfusion Injury by Improving Mitochondrial Function via Immunoproteasome-PP2A-AMPK Signalling
Source: Nutrients. 2023 Feb 20;15(4):1049. doi: 10.3390/nu15041049 (PMC9967761; doi:10.3390/nu15041049)
Supplement: Supplementary file 1 [file nutrients-15-01049-s001.zip › nutrients-2233475-supplementary.pdf]

Supplementary Materials

Supplementary Table S1. Echocardiographic measurement of wild type (WT) mice with Ursolic acid injection subjected to I/R model for 24 hours.

Parameter	Veihcle Sham	UA Sham	Veihcle I/R	UA I/R
EF(%)	69.43±1.542	69.88±1.252	26.36±0.9466**	37.14±2.397##
FS(%)	38.03±1.264	38.33±0.9937	12.08±0.4788**	17.58±1.291##
LVID;d(mm)	3.246±0.1467	3.214±0.01582	4.208±0.04445**	3.795±0.09054#
LVID;s(mm)	2.012±0.06047	1.982±0.03245	3.700±0.03834**	3.129±0.09987#
LVPW;d(mm)	1.031±0.08078	0.9504±0.09043	0.6283±0.03676**	0.9504±0.1273#
LVPW;s(mm)	1.376±0.1143	1.257±0.04870	0.8607±0.05011**	1.232±0.1352#
LVAW;d(mm)	0.9628±0.02222	0.9892±0.06842	0.6195±0.05865**	0.8448±0.07377#
LVAW;s(mm)	1.417±0.05119	1.397±0.07854	0.6935±0.1075*	1.031±0.07584#

Values are mean ± SEM, $n = 6$ per group. EF, ejection fraction; FS, fraction shortening; left ventricular mass; LVID, left ventricular internal diameter; LVPW, left ventricular posterior wall; LVAW, left ventricular anterior wall. *$p < 0.05$, **$p < 0.01$ versus Veihcle+sham; #$p < 0.05$, ##$p < 0.01$ versus Veihcle+I/R.

Supplementary Table S2. List of primary antibodies used in immunoblotting analysis.

Primary antibodies name	Company	Article Number
β1i	Abcam	ab242061
β2i	Abcam	ab183506
β5i	Abcam	ab3329
PP2A	Abcam	ab32056
AMPKα	Cell Signalling Technology	5831S
Phosphorylated (p)-AMPKα	Cell Signalling Technology	2535T
PGC1α	Proteintech	66369-AP
Drp1	Cell Signalling Technology	5391S
Mfn1	Proteintech	13798-1-AP
Mfn2	Proteintech	12186-1-AP
Ubiquitin	Proteintech	10201-2-AP
